# Access to palliative care for homeless people: complex lives, complex care

**DOI:** 10.1186/s12904-018-0368-3

**Published:** 2018-10-24

**Authors:** Anke J E de Veer, Barbara Stringer, Berno van Meijel, Renate Verkaik, Anneke L Francke

**Affiliations:** 1 Netherlands Institute for Health Services Research (Nivel), P.O. Box 1568, 3500 BN Utrecht, The Netherlands; 2 Center for Consultation and Expertise, Gouda, The Netherlands; 3 Inholland University of Applied Sciences, Amsterdam, The Netherlands; 4 University Medical Center, VU, Department of Psychiatry, Amsterdam Public Health Research Institute, Amsterdam, The Netherlands; 5 Parnassia Psychiatric Institute, The Hague, The Netherlands; 6 GGZ-VS, Academy for Masters in Advanced Nursing Practice, Amsterdam, The Netherlands; 7 University Medical Center, VU, Department of Public and Occupational Health, Amsterdam Public Health Research Institute, Amsterdam, The Netherlands; 8 Expertise Center for Palliative care, Amsterdam, The Netherlands

**Keywords:** Palliative care, People experiencing homelessness, End-of-life care, Psychiatric care, Interviews, Qualitative study

## Abstract

**Background:**

People experiencing homelessness often encounter progressive incurable somatic diseases in combination with psychiatric and psychosocial problems, and many need palliative care at the end of their lives. Little is known about how palliative care for this group can be started in good time and provided optimally. The objective of this paper is to give insight into the extent people experiencing homelessness have access to good palliative care.

**Methods:**

Qualitative in-depth interviews were held to reconstruct the cases of 19 people experiencing homelessness in the Netherlands. Eight cases concerned persons being in the palliative phase (using the surprise question) and the other 11 cases concerned persons recently died after a period of ill health due to somatic illness. We used purposive sampling until data saturation was reached. The total number of interviews was 52. All interviews were transcribed verbatim and analysed inductively.

**Results:**

Three key themes were: ‘late access’, ‘capricious trajectory’ and ‘complex care’. The first key theme refers to the often delayed start of palliative care, because of the difficulties in recognizing the need for palliative care, the ambivalence of people experiencing homelessness about accepting palliative care, and the lack of facilities with specific expertise in palliative care for them. The second key theme refers to the illness trajectory, which is often capricious because of the challenging behaviour of people experiencing homelessness, an unpredictable disease process and a system not being able to accommodate or meet their needs. The third key theme refers to the complexity of their care with regard to pain and symptom control, psychosocial and spiritual aspects, and the social network.

**Conclusions:**

The care for in the palliative phase does not satisfy the core requirements of palliative care since there are bottlenecks regarding timely identification, the social network, and the assessment and management of physical symptoms and psychosocial and spiritual care needs. Education in palliative care of outreach professionals, training staff in shelters in the provision of palliative care, and building a network of palliative care specialists for people experiencing homelessness.

## Background

The United Nations states that health facilities, goods and services must be accessible to all, especially the most vulnerable or marginalized sections of the population [1]. This also accounts for palliative care. Palliative care “improves the quality of life of patients and their families facing the problem associated with life-threatening illness, through the prevention and relief of suffering by means of early identification and impeccable assessment and treatment of pain and other problems, physical, psychosocial and spiritual.” [2] People working with the homeless, however, often have doubts about whether they have equal access to good palliative care. Palliative care facilities and expertise are often organized with the general population in mind. In the Netherlands, palliative care is preferably provided at home or, for those who need care that cannot be delivered at home, in nursing homes or hospices.

However, for people experiencing homelessness good palliative care, preferably ‘at home’, is not a matter of course. A ‘person experiencing homelessness’ is someone without permanent housing who may live on the streets, in a shelter, in temporary accommodation, or in some other unstable or non-permanent situation [3]. Based on the number of people known to the Dutch social shelter system, the number of people experiencing homelessness is estimated at 30,000, and this number is thought to be growing [4, 5]. In the Netherlands, ill people experiencing homelessness often stay in special shelters at the end of their lives, which can be seen as their ‘home’. Types of shelters, usually organized by the municipality or the Salvation Army, vary across towns. There are temporary shelters for those who are ill or frail, nursing home wards, and shelters offering hospice care.

People experiencing homelessness are a specific target group not only because of their living conditions but also because they have a short life expectancy, their high prevalence of illness and symptom burden, their concerns how their life will end, and the reduced availability of palliative care. In a cohort study in one of the largest cities in the Netherlands, life expectancy at age 30 was 11.0 (95% CI 9.1–12.9) years less for men experiencing homelessness compared to men in the general population and 15.9 (95% CI 10.3–21.5) less for women experiencing homelessness [6]. People experiencing homelessness aged 50 and older were found to have higher rates of age-related conditions (functional impairments, cognitive impairments, falls, urinary incontinence) than the general population aged 20 years older [7]. Those who do not die suddenly, for instance in an accident, by suicide, or from a drug overdose, violence or hypothermia, often need palliative care in the final phase of their life.

Heart diseases, respiratory diseases, diseases of the digestive system and alcohol-related diseases (such as liver problems or cancer) have been cited as major chronic diseases causing death among people experiencing homelessness [8–11]. Complex comorbidity may occur due to the co-occurrence of physical illness, psychiatric conditions (such as depression, psychosis, post-traumatic stress disorder, and schizophrenia) and addictions [7, 12]. When nearing the end of life, people experiencing homelessness suffer a high incidence of symptoms, especially pain, fatigue, and psychological symptoms such as worrying and feeling sad [13].

Previous studies have reported that people experiencing homelessness often have concerns about how their life will end, e.g. about dying anonymously and being undiscovered or unidentified after death [14, 15]. They also frequently worry about the funeral and notification of family members [14]. Older people experiencing homelessness often see a good death as dying peacefully, not suffering, experiencing spiritual connection, and making amends with significant others [16].

These concerns and the diversity and complexity of health problems and needs in different areas of life pose challenges to the access to good palliative care, i.e. people-centred care. Studies indicated that such care is less widely available to people experiencing homelessness [15, 17–22].

Hence the objective of this paper is to give an insight into the extent good palliative care is accessible for people experiencing homelessness in the Netherlands. Answers on this question may give a better understanding of how palliative care for this target group can be improved.

## Methods

### Design

Qualitative interviews were held with people experiencing homelessness at the end of life, as well as with people who were close to them at the end of life. In this way we tried to collect as much information about a case as possible.

### Recruitment and participants

With the surprise question (see below), we purposively recruited people experiencing homelessness, both men and women, in different living circumstances, who were at the end of their lives. We also included cases of persons who were already deceased. The interviewees and deceased cases were selected through contact persons at 13 different shelters, e.g. run by the Salvation Army or municipalities. In the Netherlands, the national healthcare policy is to have no one sleeping rough on the streets, but instead to have them sleep in night-care shelters for the homeless, for instance. Those who do not want to live on the streets anymore have access to sheltered accommodation. There are also special convalescence care shelters for sick people [23]. The shelters involved in our study were in seven cities located in different parts of the Netherlands. This diverse range of settings was selected to take into account the variety of possible palliative care circumstances, since the facilities for people experiencing homelessness can vary between municipalities.

A contact person at each shelter was asked to select cases for our interview study. They were asked to identify persons in the palliative phase with the aid of the surprise question: “Would you be surprised if this person died within the next two years as a result of his/her underlying somatic illness?”. If the answer was “No, this would not surprise me”, the contact person asked that person whether they would be willing to participate in the study and whether they gave permission to have their name and address passed on to the researcher. The contact person verbally gave information and handed the researchers’ information letter plus an informed consent form to the person. After interviewing the person experiencing homelessness, we asked their consent for also interviewing the professionals closest to them, specifically at least one professional who knew their personal circumstances (e.g. a social worker) and one professional who knew their medical circumstances (i.e. physician or nurse). Information of social workers and medical professionals supplemented each other because the nature of their contacts with the person experiencing homelessness was different. After this informed consent, we contacted the professionals for an interview.

In addition, contact persons were asked to identify cases of deceased persons who had died less than 1 year ago after a period of ill health due to a somatic illness. The contact person informed the professionals who had been most closely involved about the research and gave them an information letter with an informed consent form. After obtaining their informed consent, we contacted the professionals for an interview.

We also tried to find a family member or friend to interview (via the person experiencing homelessness or, if deceased, via the professionals). However, about half of the persons in the study cases did not have any contact with family or friends. In the other half of the cases, the relatives refused to participate or professionals were ambivalent asking them.

Participants were provided with a €20 gift voucher, participants who were homeless received €20 in cash.

### Data collection

In total 52 individual in-depth interviews were held dealing with 19 cases of people experiencing homelessness: seven interviews with people themselves (*n* = 7), 13 with social workers, 12 with physicians, 16 with registered nurses, three with nurse assistants, and one interview with a sheltered housing facility coordinator.

The interviews were almost always held in the shelter in which the person was living or had lived. The interviews with people experiencing homelessness were conducted by the first author (AdV), where another author (BS) was co-interviewer during the first three interviews. The interviewers were a psychologist (AdV) who worked in a walk-in centre for homeless people, and a mental health nurse specialized in treatment of patients with severe personality disorders (BS). Both are experienced qualitative researchers. BS – with her experience in mental health nursing - trained AdV in how to interact with people from our target group and how to develop trust. After three interviews, each extensively evaluated afterwards, AdV carried out the interviews. The interviews lasted between 20 and 90 min. Open questions were used, encouraging interviewees in their own words to describe the care the person needed and the care they received.

The interviews with the people experiencing homelessness started with a general question concerning their perceived health (“Can you tell me something about your health?”), followed by questions about the care they needed and the care they received. The first question for professionals was: “Can you tell me something about the care you give/gave?” Depending on the answer, questions were asked about the palliative care that was needed and the care provided. A topic list (Table 1) was used to see if the different aspects of palliative care were being addressed. For all groups we used the same topic guide, reminding the interviewer of possible topics to cover and areas to probe. However, during the interview this topic guide was individualized taking into account the specific situation of the interviewee and the findings from previous interviews. The primary focus was on the information the interviewee had and what the interviewee found important to tell. Because people experiencing homelessness and facing the end of their life are very vulnerable in many ways [24] the interviewers continuously paid attention to whether topics could be addressed.

**Table 1:** Topic list used in the interviews

• The care needed and the care provided (physical, ADL, psychosocial, spiritual, social, symptoms)	
• The beginning of the palliative phase	
• Proactive care	
• Communication with the person experiencing homelessness, wishes of the person	
• Autonomy of the person experiencing homelessness	
• Family and friends	
• Communication and cooperation between professionals	
• End-of-life decisions	
• After-death care	
• Care for other residents	
• Care for professionals	
• Competencies of professionals	

### Analysis

Each interview was audiotaped and transcribed verbatim. The analysis was part of a cyclical process of data collection – data analysis – new data collection, ultimately ending in data saturation [25]. Data saturation was obtained after 52 interviews based on 19 cases.

The data were analysed inductively. All transcripts were analysed by the first author (AdV), while each of the other authors analysed at least three transcripts. Initially, codes were generated that remained close to the words of the interviewees. Subsequently, the meaning of clusters of codes was discussed within the research team and key themes were identified. Transcripts of previous interviews were reanalysed and new interviews analysed, guided by these themes, which in turn were sometimes refined or reformulated during the analysis process. Interim analyses were discussed by several pairs of authors. Differences between the researchers in the analysis and interpretations were discussed until consensus was reached. Thus the coding scheme was jointly refined over time. Finally, the transcripts were coded with the final themes and subthemes, which are also used as the headings and subheadings in the Results section (see below).

Strategies to ensure rigour in our data were purposive sampling aimed to reflect the diversity in the population and multiple interviews per person experiencing homelessness aimed to capture rich, descriptive data. Interviews were analysed by the researchers from early on and after 19 cases and 52 interviews data saturation was reached.

The software program MAXQDA (release 11.0.9b) was used to facilitate the process of data analysis (www.MAXQDA.com). To validate the results, we discussed the central themes that emerged from our analyses in two focus groups with professionals and a representative of the homeless group (11 participants in total).

## Results

### Characteristics of people experiencing homelessness

Table 2 shows that the cases consisted of 16 men and three women: eight were in the palliative phase and 11 were people who were deceased. Five of the latter group died in the shelter, three in a hospice, two in a nursing home and one in a hospital. The mean age at death was 58. All had a background of homelessness, and most of them also had a history of drugs and/or alcohol abuse.

**Table 2:** Characteristics of the people experiencing homelessness (*n* = 19)

	*n*	Percentage
Person is		
- deceased	11	58%
- in palliative phase (1)	8	42%
Sex		
- male	16	84%
- female	3	16%
Age (mean, range)	Total group: 59.8 (45–72)	Deceased: 58.4 (45–70)
Problems preceding palliative phase (multiple categories possible)		
- drug addiction (including alcohol)	17	89%
- psychiatric diagnosis (e.g. schizophrenia)	5	26%
- (mild) intellectual disability	3	16%
- financial problems	1	5%
- illegal resident	2	11%
Expected or actual cause of death (multiple answers possible)		
- cancer	8	42%
- liver cirrhosis	3	16%
- COPD	3	16%
- sepsis	2	11%
- renal failure	2	11%
- cardiovascular disease	2	11%
- diabetes	1	5%

(1) One person initially agreed to participate in an interview but refused when the interviewee arrived. This person gave permission for interviews with his caregivers

### Three key themes

The interviews revealed three key themes in the provision of palliative care for people experiencing homelessness: late access to palliative care, capricious healthcare trajectories, and the high degree of complexity of the care (Table 3). These key themes were subdivided into eight subthemes. To illustrate the themes, we present the cases of Jane, John, Mitch, Anthony and William (Table 4). John and William were interviewed in the study. Jane, Mitch and Anthony were deceased at the time of the study.

**Table 3:** Map with the three key themes and eight subthemes characterizing the provision of palliative care in people with a background of homelessness

Late Access	• difficulties in recognizing need
	• ambivalence towards accepting care
	• no palliative care facilities
Capricious trajectory	• challenging behaviour
	• unpredictable disease progression
Complex care	• pain and symptom control
	• psychosocial and spiritual care
	• social network

**Table 4:** Cases illustrating the results of the analysis

Jane* was a woman without family or friends, diagnosed with schizophrenia. After a period of homelessness she lived in an assisted living facility. She died of colon cancer in a hospital aged 60. In the final two years of her life she had a small apartment and received assertive community treatment.	
*John* is an illegal resident in the Netherlands aged 52, addicted to alcohol and drugs. Because of his bad health and with the support of a street doctor, he got permission to stay in a shelter. After a hospitalization, he moved to a nursing ward for people experiencing homelessness.	
*Mitch* was a former offender, aged 56, diagnosed with an antisocial personality disorder and long-lasting and severe drugs abuse. He was a drugs dealer and had received several convictions for serious violent crimes. He had been living for some years with his wife in his own apartment and had been receiving assertive community treatment. He died in a hospice of oesophageal cancer.	
*Anthony* was born with a mild intellectual disorder and was addicted to alcohol. Lived in a shelter for people with addiction problems or severe psychiatric problems with somatic complaints. Remarkably, Anthony still had close ties with his family: every day they met each other in his mother’s home to play games and drink beer. He died of throat cancer at the age of 53 in a hospice.	
*William* is a friendly man aged 52. He is addicted to alcohol and drugs and his aggression regulation is distorted. He suffers from COPD and is oxygen dependent. He lives in a shelter for people experiencing homelessness with somatic health problems. * Names are pseudonyms and bear no resemblance of those interviewed	

#### Late access

People experiencing homelessness were found to have late access to palliative care. Three subthemes were factors in this late access: difficulty among professionals in recognizing palliative care needs, the ambivalence felt by them towards searching for and accepting care, and the lack of services specialized in palliative care for people experiencing homelessness.

##### Difficulty in recognizing palliative care needs

People experiencing homelessness often do not have a recorded medical history due to their often chaotic lifestyles and the paths their lives have taken. Earlier somatic problems were often not documented, which made current signals difficult to interpret. Interpretation was also difficult because people experiencing homelessness mostly had a combination of psychiatric symptoms, addiction problems, intellectual disabilities and challenging behaviours. A social worker: *“There is not much continuity in my relationships with people experiencing homelessness. They come and go again. This makes it extra difficult to notice changes in health care needs.”*

The professionals involved were often social workers who were not trained in recognizing medical problems and potential palliative care needs. As Jane’s case illustrates, for most professionals it was difficult to recognize that someone was incurably somatically ill and in need of palliative care (Table 5, E1).

**Table 5:** Examples (indicated as E1 to E8) illustrating the characteristics of palliative care provision

Subtheme	No.	Quote
Difficulty in recognizing need	E1	Jane’s social worker described their search: *“Jane complained about her stomach and intestines… we thought that was part of her psychiatric illness. In retrospect I think these were the first signs of cancer….We arranged admission to a psychiatric hospital… a forced admission because she refused to go voluntarily. If you work in psychiatry, you sometimes focus too much on mental issues, you forget that it may just be something somatic.”* In the psychiatric hospital no further medical examinations were done. After discharge from the psychiatric hospital, the social workers noticed that Jane was rapidly losing weight. Because Jane had not visited a GP for years and occasionally made remarks about hospital experiences, the social workers contacted all the hospitals in the region to search for more medical information, but no file on Jane was found. Gradually they realized that Jane was probably very ill. Her social worker*: “If there had been a medical file, we might have spotted signs of digestive problems earlier”*.
Ambivalence towards accepting care	E2	A nurse talking about Mitch: *“I persist in motivating him to come to our yearly somatic screening, for instance, but some will never come… Every time, we try to make contact with a person again. We can be very persistent. Even when I see someone on the street or in a shop I talk to him, both during working hours and during my free time.”*
No palliative care facilities	E3	A nurse at Anthony’s shelter: “*I work in this shelter two days a week, mainly to arrange all kinds of things. … We can do a lot, that’s not the problem. Anthony wants to stay here and, however fragile, some of us sometimes have a little talk with Anthony. But there are different points of views in the team as to how far we can go in offering care for the dying… In this shelter there are no nurses or nurse assistants. But Anthony didn’t want to leave and, besides, what nursing home will tolerate his behaviour?”*
Challenging behaviour	E4	The nurse involved with John explained why John had not yet transferred to a nursing home, despite his care needs which they could not meet properly: “*John can’t go to the nursing home yet. He won’t stick to the rules. He will persist in smoking in his room. In the past someone was refused care in the nursing home because he persisted in smoking, despite warnings. Some nurses didn’t dare enter the room because they were afraid of him. Therefor John will only be transferred to the nursing home when this behaviour has faded…so, for the final days of his life.”*
Unpredictable disease progression	E5	John’s nurse: *“..we thought…this will not last long. But one way or another, he recovered again. He was admitted to hospital and we thought this is the end. But then...suddenly…after two days he returned and was on the streets to take his heroin. As if he rose from the dead … I’ll never forget his will to live.”*
Pain and symptom control	E6	A social worker: *“Anthony persisted in smoking. The pain medication made him muzzy. Even when he couldn’t leave his bed anymore he still wanted to smoke. Because we were afraid he might fall asleep, some member of staff had to go to his room to let him smoke. But often we don’t have enough time to sit there several times a day. Then it is either medication, or smoking…”*
Psychosocial and spiritual care	E7	William, who had to stay in his room because of a lack of mobile oxygen: “*I would like to arrange some things, the funeral, that I have a bit of certainty… It’s just a surprise to me now. It is in the surprise package. [Laughing] [I: Did you ask for this?] I don’t know. No, well, I’ve mentioned it here a few times… but yes ... that would be nice. They know I’m very ill ... No one has come to me yet, what do you want when you’re dead, what should happen? [I: What do you want] Well, for now I want a mobility scooter ...Now it’s still possible, so I can meet some people.”*
Social network	E8	Mitch’s GP: *“Of course they clearly missed something [tumour] on the CT. If there had been relatives with him, I’m sure the urologist would have said ‘Well, gosh, what’s up there…?’ I‘ll ask... but yes up to a certain point, of course. It’s because you’re not so emotionally connected to someone. But I often get this kind of thing, that somebody takes less of a serious look at someone. Right, it shouldn’t be that way, but it is.”*

##### Ambivalence towards accepting care

People experiencing homelessness did not readily ask for help, even when they experienced healthcare needs. They had often suffered many losses in the past (e.g. their job, home, family and friends, dreams and hopes, and now their health), suffered from social exclusion, and had learned to solve things themselves. They often had no confidence in professionals due to previous disappointing contacts, feelings of not being treated equally, perceived stigmatization and being disparaged by professionals. Feelings of shame, for instance because of physical neglect or persistence in addiction, were also mentioned as a barrier to seeking help. The maintenance of autonomy and self-control was generally very important for them. From this perspective they did not easily ask for help, nor did they easily accept help, even if they had a strong need for help. Members of an assertive community treatment (ACT) team said that it was difficult to remain in touch with people experiencing homelessness. Despite this difficulty, the ACT team members said that they did not abandon the person (Table 5, E2).

##### No palliative care facilities

In the Netherlands, there are a limited number of nursing-home facilities with specific expertise in palliative care for people experiencing homelessness. However, in most parts of the Netherlands there is insufficient capacity in specialized nursing homes for people experiencing homelessness and so these people depend on mainstream care, for instance in hospitals, nursing homes, hospice facilities or shelters. Just as people experiencing homelessness are ambivalent about accepting care, so mainstream care facilities are also ambivalent about providing care for them, or even reluctant to do so. If a person failed to show up to an appointment in a hospital, for instance, the physician generally did not undertake further initiatives and in the worst-case scenario they just closed the file. Nursing homes and hospices had difficulty with their way of life, especially if they persisted in their addiction. People experiencing homelessness were generally not willing or able to make changes in their way of life, such as refraining from drugs.

At the same time, shelters for people experiencing homelessness were not able to offer palliative care. Social workers were not trained in providing the nursing care, or even the ADL care that is often needed at the end of life. As a social worker put it: *“We always focus on resocialisation, resocialisation, and resocialisation…, whereas another strategy is needed.”* Shelters for convalescent care primarily provided ADL care; other nursing care was not available 24/7. Many professionals in shelters thought that palliative care and terminal care were the same, not realizing that many of their clients were in need of palliative care even though they were not in the terminal stage of life. But the professionals in the shelters were very skilled in providing a safe environment and trusting relationships for and with their clients. If the person became sicker, professionals struggled with the dilemma of whether the client could stay or should go to a mainstream palliative care facility (Table 5, E3).

#### Capricious trajectory

Access to palliative care facilities was often impeded by big fluctuations in care provision. Two subthemes underlie these fluctuations. Firstly, the behaviour of people experiencing homelessness presents a challenge for a continuous, workable relationship with professionals. Secondly, professionals experienced the disease progression as unpredictable.

##### Behaviour that is challenging for professionals

Many of the professionals interviewed, both in facilities for people experiencing homelessness and professionals elsewhere, mentioned that the behaviour of people experiencing homelessness is often challenging and difficult for them to handle. If addicted, they spent a great deal of their time arranging and using alcohol or drugs. Social contacts were mainly based on drugs use. This instrumental behaviour was experienced as often impeding genuine social contacts and communication with professionals. The professionals used a variety of labels when describing frequently occurring difficult behaviours of people experiencing homelessness: aggressive, rejecting, passive, manipulative, norm-breaking and rule-breaking, and an associated loss of decorum (Table 5, E4).

Intellectual and cognitive disabilities, sometimes congenital and sometimes resulting from long-term addiction, further complicated communication and effective interactions. Some had schizophrenia, psychoses or other psychiatric disorders hindering communication. These challenges for professionals threatened the continuity of the care and of the relationship with the professional. Professionals invested a lot of energy in ensuring continuity in medical care by reminding people of their appointments with physicians and accompanying them to the hospital, despite the challenging behaviour they exhibited. The ACT nurse about Mitch’s contacts with the medical specialist in the hospital: *“He behaved very pettishly, very badly. He let happen what had to happen, but he was absolutely condescending… He could not accept authority at all.”*

##### Unpredictable disease progression

The state of physical health often stabilized or even got better when the sick person moved from living on the streets into a shelter. There, the person not only received somatic care but was also supported in having a regular structure to their day, a day-night rhythm and better food intake, and they received personal attention. The person was also supported in their adherence to the medical treatment. As a consequence, the person’s condition could improve more than would have been expected if the sick person had continued in their normal living conditions.

The fact that the temporary improvement was unexpected was also due to a lack of knowledge among professionals, for instance about the course of COPD. If the person had recovered from an exacerbation, many professionals interpreted the improvement as genuine recovery, not realizing that this process of recurring exacerbations characterizes COPD.

Finally, it was also difficult for professionals to get good insight into the disease progression because a person experiencing homelessness often wanted to pick up their usual way of life as quickly as possible. If, after a deterioration, the health status improved slightly, the person often tended to go back to living on the streets. The professionals said people experiencing homelessness had learned to survive from early on in life and did not easily give up. Sometimes their craving for drugs made them leave the shelter or hospital as soon as possible (Table 5, E5). These circumstances made it particularly difficult for professionals to predict the disease progression.

#### Complex care

The third key theme that prevents people experiencing homelessness from having access to good palliative care is the complexity of this care. In particular the control of pain and other symptoms, the psychosocial and spiritual care, and the care for relatives were perceived as difficult.

##### Pain and symptom control

Many of the professionals interviewed experienced difficulties with pain management and control. Recommendations in general palliative care guidelines or protocols often do not take into account the specific characteristics of persons who use or have used cocaine, methadone, mood stabilizers or antidepressants. People who already use or have used these drugs were found to react differently to the usual doses of pain medication. Some people got suboptimal pain control because professionals believed higher doses might increase the risk of premature death (Table 5, E6).

End-of-life decisions such as palliative sedation also turned out to be different, i.e. the prescribed doses did not have the expected effects due to a long history of drug use. Physicians involved in the health care for residents in shelters sometimes noticed that pharmacists and psychiatrists could not give them appropriate advice either and this made them insecure and reticent about giving higher doses of pain medication than usually recommended. Anaesthesiologists in hospitals were sometimes approached as consultants able to give useful advice in the event of problems with pain control.

In addition to the control of pain, psychological symptoms were also seen as difficult to control. In the case of psychological symptoms such as anxiety and depression, this was often due to the difficulties the people experiencing homelessness had communicating their feelings. ‘Self-medication’ (the use of alcohol or drugs) made it more difficult to take a satisfactory decision about what should best be prescribed to control the symptoms. A lack of knowledge about what the proper treatment is in the case of long-term use of all kinds of medicines in the past further hindered symptom control.

The interviewees also mentioned dyspnoea as a frequently occurring symptom that was difficult to control. For example, many people experiencing homelessness suffer from COPD and frequently have shortness of breath with regular exacerbations in COPD symptoms. They are very anxious about choking but also experience suboptimal symptom treatment. Smoking when oxygen dependent was sometimes considered as an unacceptably high fire risk in the shelter. The policy then was the suboptimal treatment of low levels of oxygen in the blood.

##### Psychosocial and spiritual care

Except for the shelters run by the Salvation Army, additional psychosocial and spiritual palliative care was not always available in the shelters. Professionals often did not know that psychosocial and spiritual care is also part of palliative care. Some professionals were afraid to talk about dying with the person experiencing homelessness. Others said that they sometimes tried to speak to the person about the fact that they were going to die, but that many people experiencing homelessness avoided having that conversation. The explanation they gave was that talking about the past and meaningful events in their lives could bring back memories that were too painful. Just ‘being there’ for the sick person was seen as the most important thing, trying to grab an opportunity to talk about it. They tried to indulge the person with attention, comfort, good food, sweets and kindness. Psychosocial and spiritual care was sometimes particularly difficult at the end of life when the person was too ill to leave the room and spend time in a common room. Professionals in shelters said that they usually did not have enough time to go to the person’s room to talk with that person, other than bringing them meals and offering physical care. Therefore most of the time the person was all alone in their room (Table 5, E7).

##### Social network

The social network of people experiencing homelessness often only consisted of other people experiencing homelessness with whom they used to drink or use drugs. If someone got sick, these contacts usually faded. Family ties were mostly broken due to painful events in the past. As William told about his relationship with his sister: *“I have neglected her entire life. I only called her when I was in jail. Then I ordered her to buy a game computer…that kind of things.”* It usually cost a lot of time to find an address and restore family ties. Renewed relationships were often painful and fragile, and needed continuous supervision so that the relationship did not break again. In most cases there were no family or friends available who could help the person experiencing homelessness to discuss complaints with medical specialists and to increase the chance of receiving good palliative care (Table 5, E8). The lack of a social network also increased the chance of dying without the presence of the immediate family or other relatives.

## Discussion

The main aim of this paper was to give insight into whether people experiencing homelessness in the Netherlands have access to good palliative care. We must conclude that there are some serious barriers that make good palliative care for this target group particularly challenging. These barriers can be clustered into three key themes: ‘late access’, ‘capricious trajectory’ and ‘complex care’.

The first theme refers to the often limited use of palliative care because of the difficulties in recognizing the need for palliative care, the ambivalence people experiencing homelessness towards accepting palliative care, and the lack of facilities with specific expertise in palliative care for the homeless.

The difficulties in recognizing a need for palliative care in this target group as identified in our study might be related to the phenomenon of diagnostic overshadowing, which is often seen in the care for people with a mental illness [26]. The person’s physical symptoms are misattributed to their mental illness, which leads to a worse physical condition.

Ambivalence towards accepting care also increases the chance of worse care. We found that this ambivalence was related to perceptions that people experiencing homelessness often have of being stigmatized. Other research shows that these perceptions are based on frequent actual prejudice and stigmatizing attitudes in services [27, 28]. The ambivalence seems to result from an interplay between the person experiencing homelessness and the social and health care services. This phenomenon in the system is also described as ‘care paralysis’, referring to the inability to help clients with multiple and complex problems by social services and care facilities [29]. Care avoidance and care paralysis can be considered as reinforcing and recalling each other. Late access to palliative care also relates to our finding that mainstream care facilities in the Netherlands often lack expertise in care for people experiencing homelessness while shelters often lack expertise and facilities regarding palliative care. An international review of studies showed that in other countries comparable challenges lie in providing good palliative care in for the target group both in hostels for people experiencing homelessness and in mainstream healthcare systems [30].

The second theme refers to the illness trajectory, which is often capricious because of the challenging behaviour of people and the unpredictable disease process. The illness trajectories of people experiencing homelessness were often found to be difficult to predict for a number of reasons, not least because care in a shelter, after having lived on the streets, might temporarily improve health. This phenomenon makes it more difficult to arrive at a proper estimation of the evolving healthcare needs of the person in question. Previous research [31] showing significant improvements in health status after a person enters the shelter system, confirms these observations. The capricious illness trajectories were also described in other research as a barrier for palliative care discussions [32].

In addition, we found that maintenance of autonomy and self-control was generally very important for the target group, which often resulted in an avoidance of care, non-cooperative behaviour and subsequently late access to palliative care. Professionals might face a dilemma in how to support the person experiencing homelessness in maintaining their autonomy: whether no further insistence to accept care or to continue to discuss the situation and the need for care with them. No further insistence respects their wish to avoid care, but ongoing discussion of the person’s situation and needs provides an opportunity for advance care planning and puts the person in control of the future situation. Although the effectiveness of existing advance care planning interventions, such as completing advance directives, has not yet been fully proven for the population of people experiencing homelessness [33], planning may help the person maintain autonomy and self-control. Further research should be done on how this dilemma can best be handled. Care providers who establish contact and win trust employ ‘non-judgemental appreciation’. They show empathy, accept what is and try to connect with the client and their world [28, 34].

The third major theme identified in our study refers to the complexity of pain and symptom control, psychosocial and spiritual care, and the lack of involvement of relatives. People experiencing homelessness frequently have complex care needs, and multiple problems and symptoms that are difficult to recognize and interpret. For instance, persons who are homeless often haven psychiatric disorders, such as schizophrenia. One of the characteristics of people with schizophrenia is that they often express lower intensity of pain when compared to other people [35]. Besides, pain people experiencing homelessness is often difficult to identify and control because of drug abuse. In addition, it is often difficult to control dyspnoea because many of these people want to carry on smoking, giving a risk of fire when oxygen is used. This may cause undertreatment.

The care is also complex because of difficulties to involve relatives. Whereas in the general population relatives are often directly involved in palliative care, in the homeless population the family is often absent, also at the end of life. Supporting individuals experiencing homelessness in restoring family bonds is an exceptionally challenging task for professionals. The absence of family is also illustrated by the fact that we were not able to involve immediate family or other relatives, either because they had their own problems or because they did not want to resurrect the past. The lack of a social network and the ambivalence felt by professionals about contacting the immediate family or other relatives are also found in other research among people experiencing homelessness [14, 15, 36].

In addition, we found that providing good psychosocial and spiritual care is often difficult. Professionals sometimes did not know that this kind of care is also part of palliative care, were afraid of talking about dying, or did not have enough time for it. People experiencing homelessness also often avoid such conservations. Our study however also showed that some wanted to talk to someone else about psychosocial and spiritual issues, such as reflecting about their life and the near future. This is in accordance with studies showing that people experiencing homelessness do worry about the end of life [11–13]. Respect for the person is a precondition for developing trust and improving the engagement with them, which is needed for tailored psychosocial and spiritual care [36].

### Strengths and limitations

A strength of this study is the multi-perspective approach: the cases of 19 people experiencing homelessness in need of palliative care were described incorporating information of all the people most closely involved. A second strength is that we selected a variety of cases of people experiencing homelessness, receiving care from a variety of services. This variety contributed to the validity of the results.

A limitation is that no immediate family members or other relatives were interviewed. Family members refused, mainly because the memories were too painful and/or they wanted to close the book on this. Often, there were no contact details for family members. Underlying processes and how to deal with them are worthwhile studying.

### Practice implications

Late access to palliative care, a capricious illness trajectory and the complexity of care have consequences for professionals involved in the care for people experiencing homelessness at the end of life.

Based on the results we recommend the following for timely, good palliative care for the homeless:

- Informing outreach professionals, such as street workers, about palliative care. They can play an important role they can play in signalling, documenting and reporting whenever they suspect that someone is seriously ill. People experiencing homelessness themselves or those who were formerly experiencing homelessness can easily make contact with other people experiencing homelessness. They may also have a role in signalling a need for palliative care.
- Training staff in shelters in palliative care and the ways palliative care can be organized is a first step towards improving palliative care for people experiencing homelessness living in shelters. Staff in mainstream palliative care facilities (nursing homes, hospices) can be trained and supported to deal with clients with challenging behaviour and the special needs they may have (e.g. regarding addiction, pain and symptom control, and involvement of family and friends).
- Building a network of palliative care specialists for people experiencing homelessness, who can advise professionals in mainstream facilities, e.g. regarding the complexities of pain and symptom management in this target group. This can stimulate growth of the body of knowledge about palliative care for people who are homeless.

There is some evidence that interventions aimed at advance care planning, documenting wishes, and better cooperation may be a potentially effective ways to encounter the concerns and needs of homeless people in the final phase of their life [14, 17, 33]. However, further research is needed on the effectiveness of these interventions.

## Conclusion

According to the definition of the World Health Organization [2], palliative care is “an approach that improves the quality of life of homeless individuals and their families facing the problem associated with life-threatening illness, through the prevention and relief of suffering by means of early identification and impeccable assessment and treatment of pain and other problems, physical, psychosocial and spiritual.” This study shows that people experiencing homelessness have less access to this care. The absence of a medical record, the lack of palliative care expertise and ambivalence of people who are homeless to ask for and accept care make timely identification of a need for palliative care difficult. Complex social relationships and no or fragile family bonds make involvement of the social network challenging. Assessment and treatment of pain and other problems is complicated by in particular the ambivalence towards accepting care, challenging behaviour, and (former) use of alcohol or drugs. We conclude that the palliative care currently provided to the people experiencing homelessness does not meet the core requirements of palliative care due to bottlenecks regarding timely identification, care for the social network, and the assessment and management of physical symptoms and psychosocial and spiritual care needs.

## Abbreviations

ACT: assertive community treatment
ADL: Activities of Daily Living
CI: Confidence Interval
COPD: Chronic Obstructive Pulmonary Disease
FNO: Fonds Nuts Ohra
RCOAK: Roomsch Catholijk Oude Armen Kantoor
WHO: World Health Organization
